# A Case Report on Vaginal Melanoma That Metastasized Distantly

**DOI:** 10.7759/cureus.63069

**Published:** 2024-06-24

**Authors:** Sudhanshu Tonpe, Himandri Warbhe, Pankaj Banode, Sneha Bandi, Jignesh Kumar Patel

**Affiliations:** 1 Department of Interventional Radiology, Jawaharlal Nehru Medical College, Datta Meghe Institute of Higher Education and Research, Wardha, IND; 2 Department of Respiratory Medicine, Jawaharlal Nehru Medical College, Datta Meghe Institute of Higher Education and Research, Wardha, IND; 3 Department of Radiology, Siddhartha Medical College, Vijayawada, IND; 4 Department of Pulmonary Medicine, PD Hinduja National Hospital & Medical Research Centre, Mumbai, IND

**Keywords:** ct, mri, postmenopausal woman, vaginal tumor, vaginal melanoma

## Abstract

A case report of a 55-year-old woman who had just gone through menopause complained for a month about objects coming out of her vagina with a foul-smelling vaginal discharge. A 3-4 cm tumor growing from the vagina was discovered on a vaginal examination. The growth bled on contact and was friable. The patient also complained of multiple lumps on the body and difficulty in breathing. The patient underwent pelvic magnetic resonance imaging (MRI) and computed tomography (CT) of the chest and was diagnosed with vaginal melanoma with distant metastasis. Following radiotherapy, a sizeable local excision of the vaginal masse was done as a palliative measure, along with the dissection of both inguinal lymph nodes. After experiencing abrupt dyspnea six months prior, the patient's CT scan of her chest showed the growth of metastatic lesions in her lungs, and she eventually passed away from her illness.

## Introduction

Malignant melanoma is a common and dangerous skin cancer that can develop in the mucous membranes of the genitourinary, respiratory, and gastrointestinal tract. These uncommon mucosal melanomas, which account for 1% of all melanomas, are derived from the melanocytes in the mucous membranes [1]. Malignant melanoma seldom occurs in the female vaginal canal. In the female genitalia, the vulva and vagina are the most often affected areas by malignant melanoma. Merely 3% of melanomas in the female genitalia are vaginal melanomas. In females, this amounts to 0.3% to 0.8% of all melanomas. There are only 250 instances of vaginal melanoma reported in the literature to date, and the incidence is just 0.46 cases per one million women annually [2,3]. The five-year survival rate for vaginal melanoma is a pitiful 5%-25% [2]. Since vaginal melanoma is extremely uncommon, we report a case study together with a literature review.

## Case presentation

A 55-year-old woman who had just gone through menopause complained primarily of vaginal discharge and something coming out of her vagina every month. During per vaginal examination, a 3-4 cm tumor developing from the posterior vaginal wall was found. The lesion bled on contact and was friable. In addition, the woman reported having trouble breathing and having several tumors on her body. The patient had a chest computed tomography (CT) scan and a pelvic magnetic resonance imaging (MRI).

**Figure 1:**
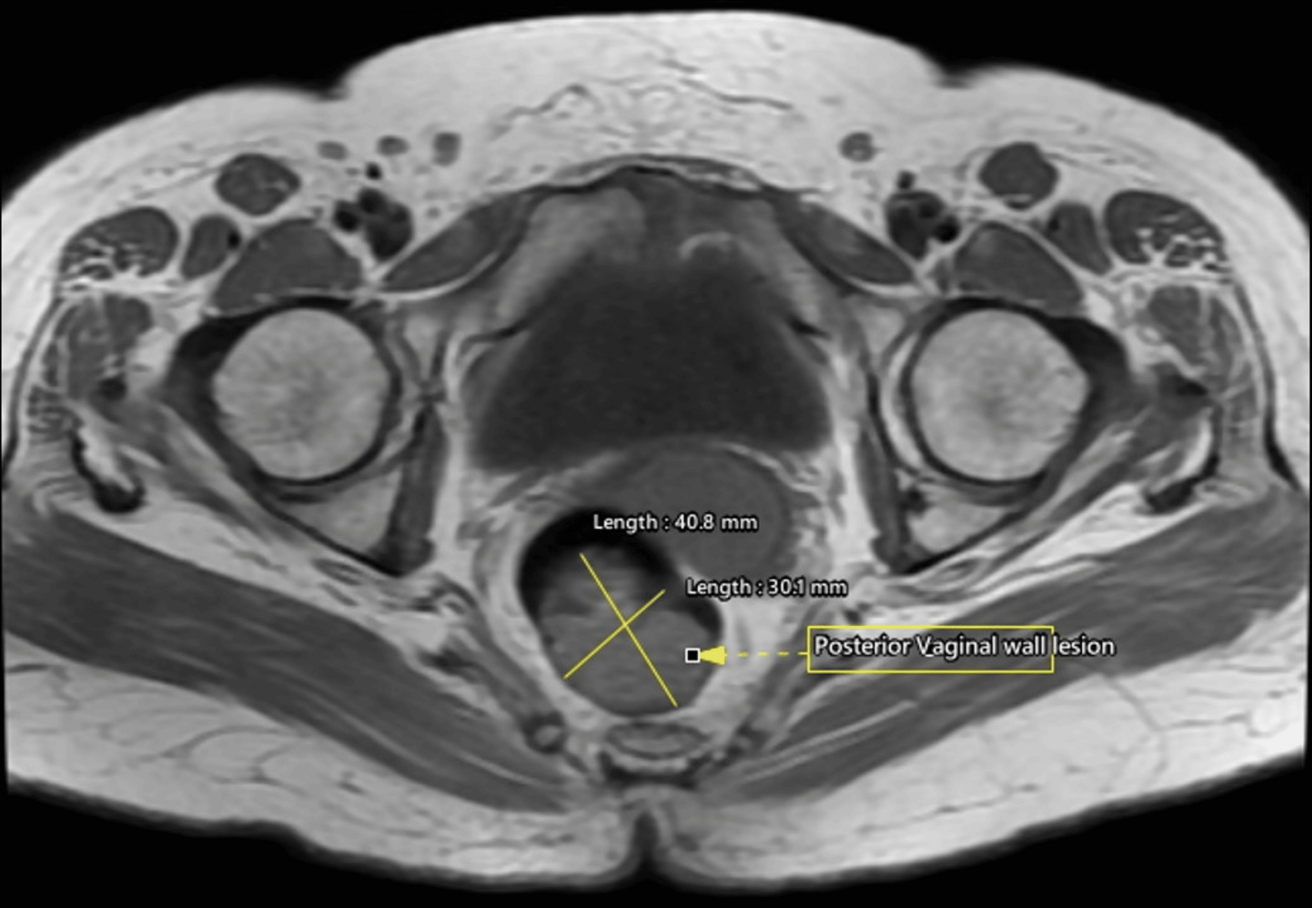
Magnetic resonance imaging (MRI) axial T1 images of the pelvis at the level of the vagina demonstrating a well-defined iso- to hyperintense lesion along the posterior wall of the vagina (yellow arrow)

**Figure 2:**
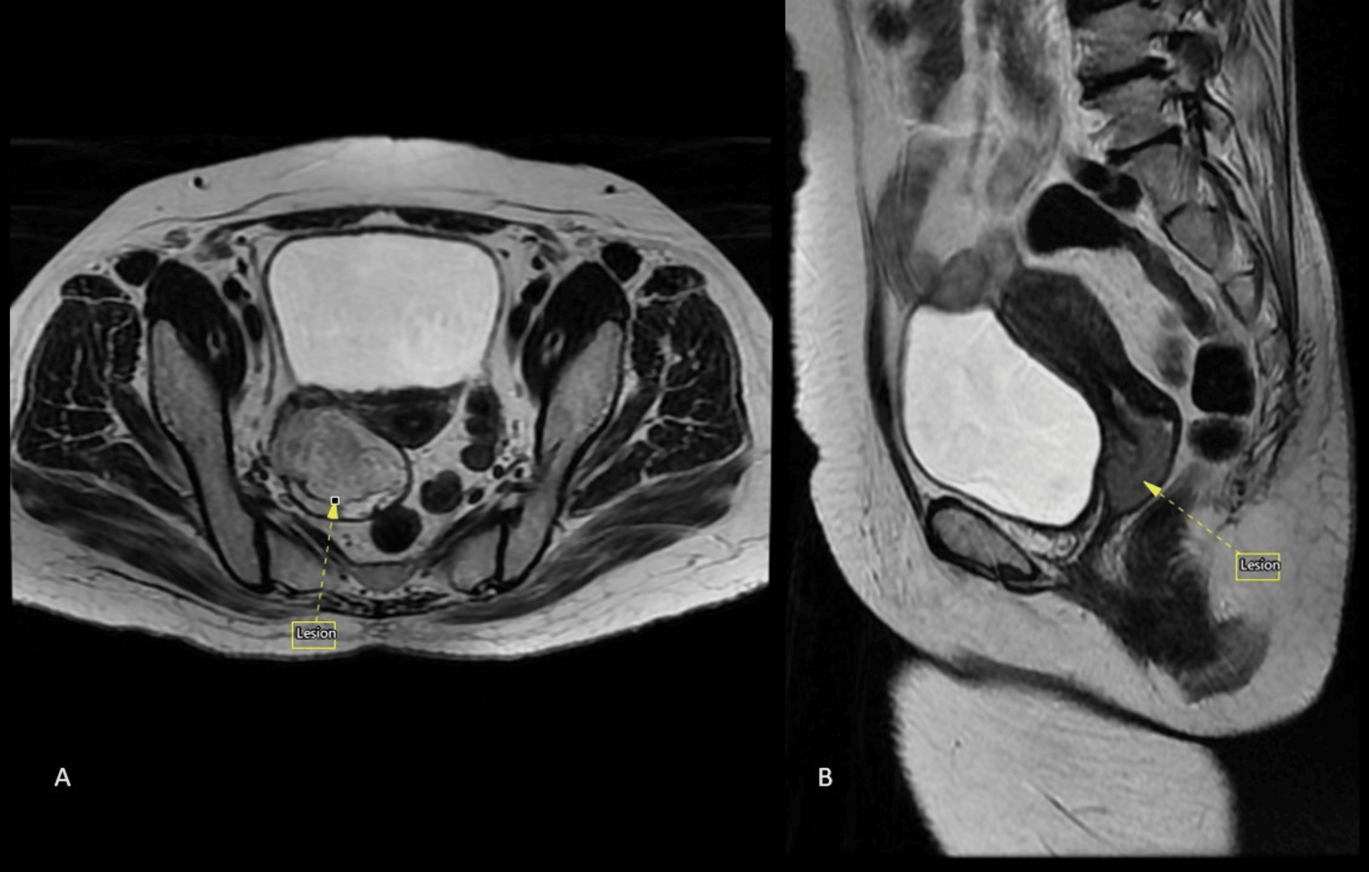
Magnetic resonance imaging (MRI) T2 axial (A) and sagittal (B) sections at the level of vagina shows a well-defined heterogeneous T2 hyperintense lesion originating from the posterior vaginal wall

**Figure 3:**
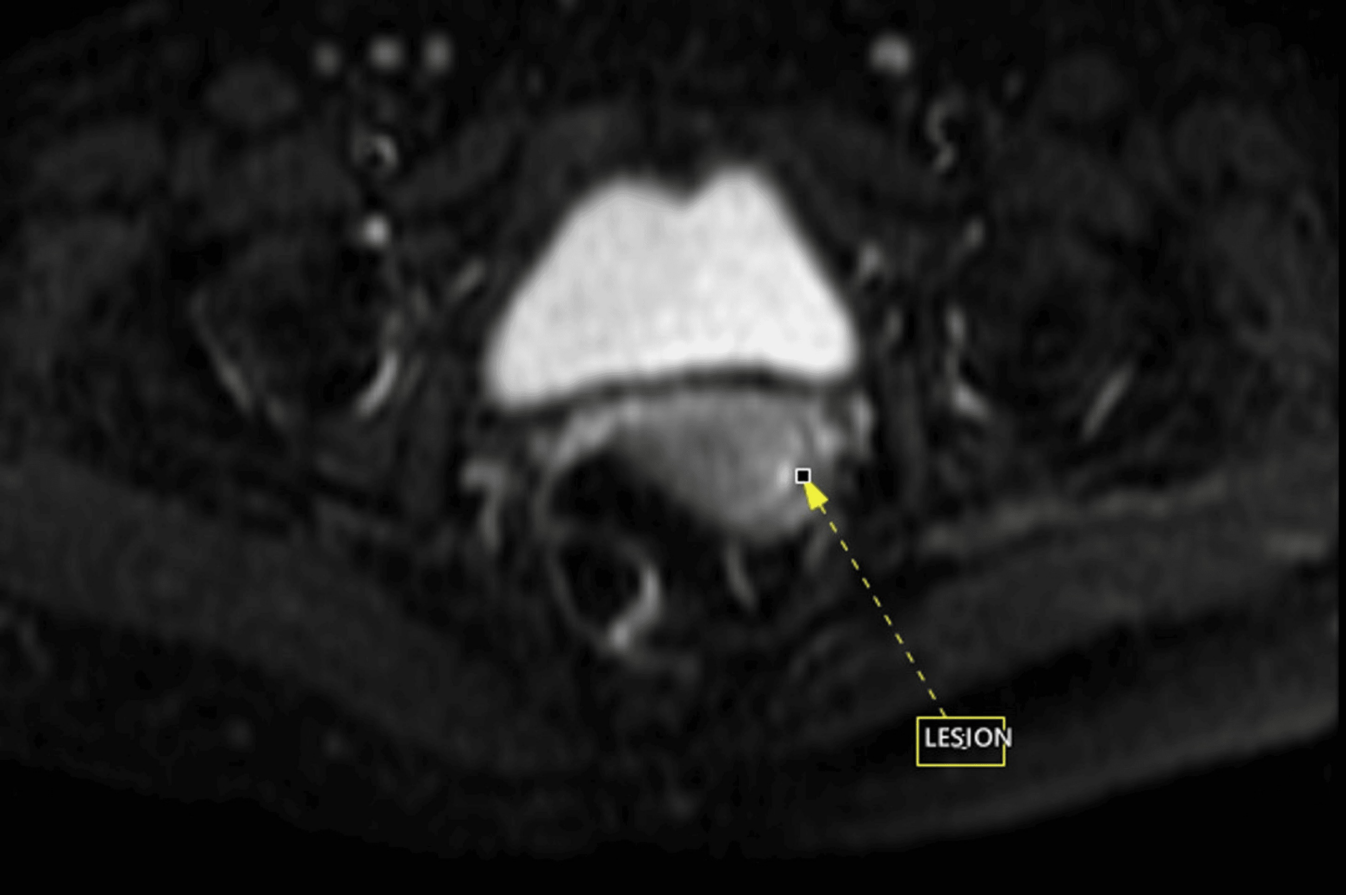
Diffusion-weighted imaging (DWI) sequence magnetic resonance imaging (MRI) at the level of the vagina shows lesion in the vaginal vault showing diffusion restriction

A 4 cm by 3 cm mass was detected via an MRI scan; the lesion was iso- to hyperintense intense on T1-weighted sequences and hyperintense signal on T2-weighted sequences and diffusion restriction on diffusion-weighted imaging (DWI) sequence (Figures 1-3).

**Figure 4:**
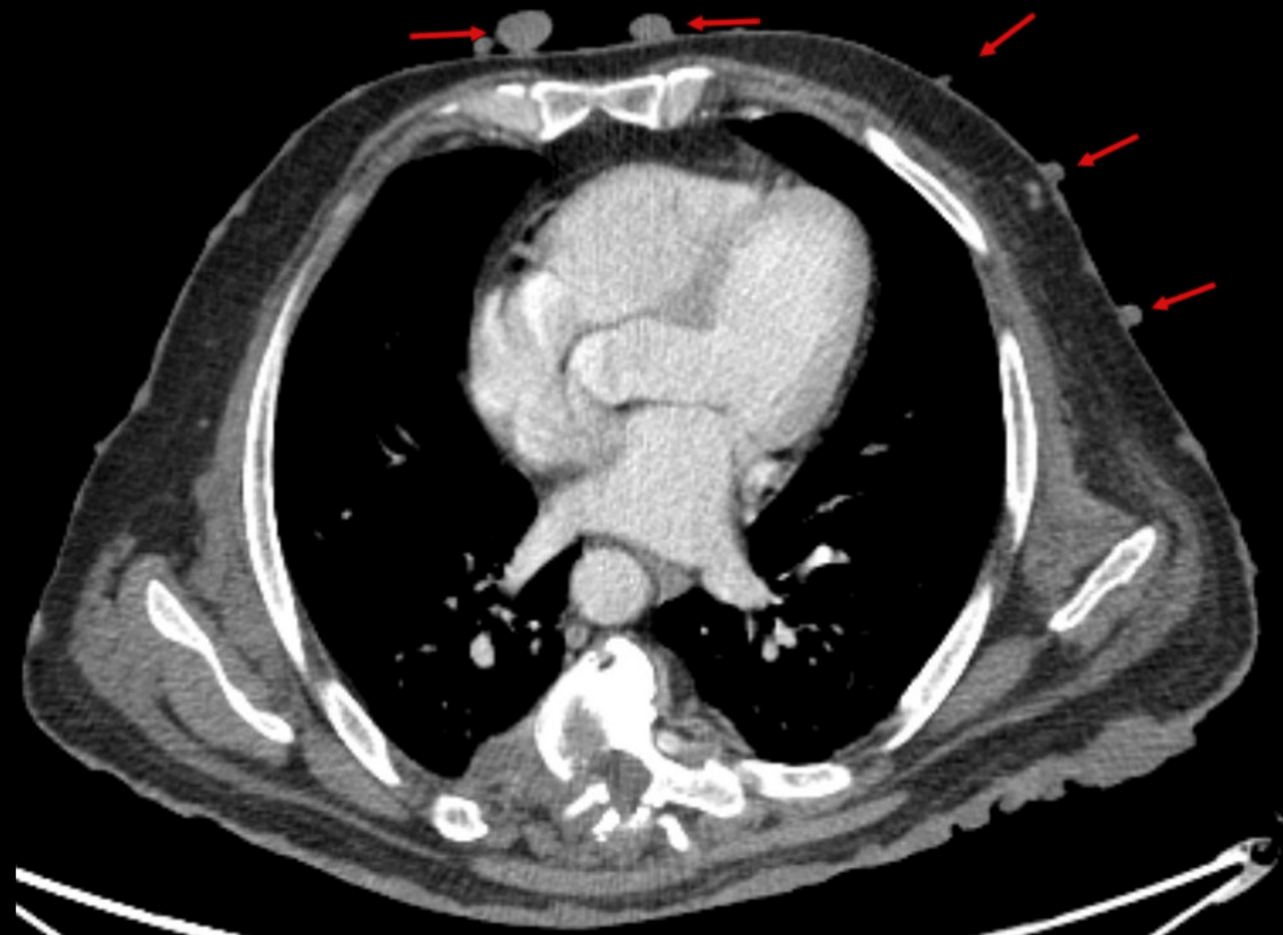
Axial sections of the contrast-enhanced computed tomography (CECT) chest in soft tissue window at the level of the heart showing multiple percutaneous enhancing soft tissue lesions (red arrows)

**Figure 5:**
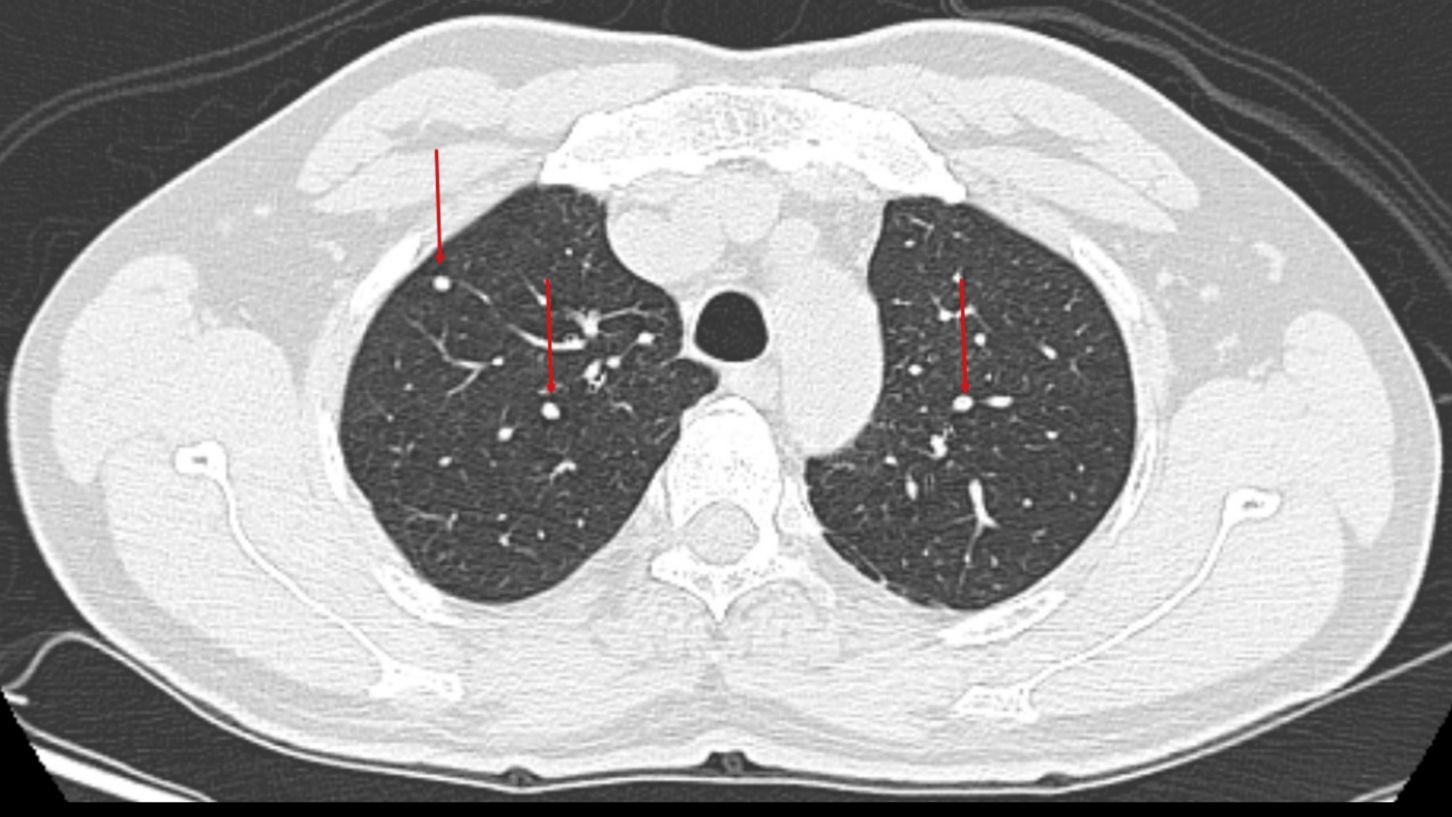
Axial sections of the contrast-enhanced computed tomography (CECT) chest in lung window at the level of the aortic arch showing intraparenchymal pulmonary nodules (red arrows)

**Figure 6:**
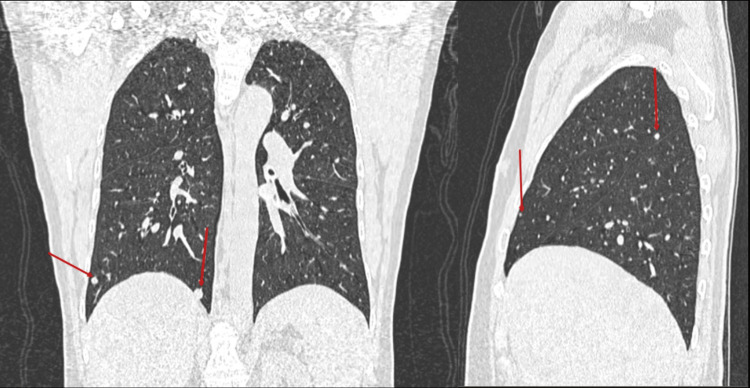
Contrast-enhanced computed tomography (CECT) of the chest in lung window in coronal and sagittal reformats at the level of the aortic arch showing Intraparenchymal pulmonary nodules (red arrows)

The patient's contrast-enhanced computed tomography (CECT) scan revealed several soft tissue density lesions in the back and chest wall as well as multiple heterogeneously enhancing masses of varying sizes in the lungs (Figures 4-6).

After the vaginal lesion biopsy, a diagnosis of vaginal melanoma with distant metastases was made. A palliative-wide local excision of the vaginal masse with bilateral inguinal lymph nodal dissection was performed, followed by radiotherapy. After seven months, the patient presented with a sudden onset of breathlessness, and a CT scan of the chest revealed extensive metastasis, and the patient succumbed to her disease.

## Discussion

Only 3% of vaginal malignancies are vaginal melanoma, an uncommon and highly deadly cancer [1]. Melanocytes found in the vaginal epithelium's basal layer are the source of it [4]. When symptoms like pain, vaginal discharge, vaginal bleeding, and vaginal mass are present, the lower portion of the anterior vaginal wall is the most common location [5]. Because of its distant metastases, recurrence, and local expansions, it is the most hazardous type of vaginal tumor [6]. The lamina propria of the vaginal mucous membranes receives a substantial lymphatic and vascular supply, which causes extensions and metastases [7]. The groin, vulva, and vagina are the typical recurrence locations [8]. The lymphatic drainage of the vaginal mucosa follows the lymphatic channels and nodes. Cancers in the higher third spread to the standard and internal iliac nodes, the middle third to the superficial inguinal and perirectal nodes, and the lower third to the external iliac nodes [9].

Lungs are the most often affected regions of distant metastasis, followed by the liver, bones, and brain [4]. The tumor size of <3 cm is a good prognostic factor, but tumor thickness is only marginally predictive of survival [10]. Immunohistochemistry and histology are typically used to make the diagnosis [11].

The radiologic examination is crucial for prognosis, operational planning, and staging. Instead of being employed for initial staging, CT and positron emission tomography are utilized to identify distant metastases [11]. The treatment modalities most accepted are surgery and postoperative radiotherapy. Standardized and comprehensive documenting of histologic and clinical results is necessary to progress the field and make it easier to combine cases from other institutions to draw conclusions that are more statistically sound. Melanoma is more likely than squamous carcinoma to spread to other parts of the body.

## Conclusions

Finally, we report a unique instance of widespread metastases resulting from an advanced-stage vaginal melanoma. The quality of life improved dramatically when all visible tumors were surgically removed. Unfortunately, the patient's lung metastasis worsened in our instance, and she eventually passed away from the illness. Most information on vulvar melanoma is derived from case studies and tiny retrospective series. Documenting clinical and histologic results needs to be standardized and organized to promote knowledge advancement and make it easier to diagnose and treat such disease conditions.
